# Post-Exposure Protection in Mice against Sudan Virus by a Two Antibody Cocktail

**DOI:** 10.3390/v10060286

**Published:** 2018-05-26

**Authors:** Jeffrey W. Froude, Andrew S. Herbert, Thibaut Pelat, Sebastian Miethe, Samantha E. Zak, Jennifer M. Brannan, Russell R. Bakken, Alexander R. Steiner, Gang Yin, Trevor J. Hallam, Aaron K. Sato, Michael Hust, Philippe Thullier, John M. Dye

**Affiliations:** 1 US Army Medical Research Institute for Infectious Disease (USAMRIID), Fort Detrick, MD 21702, USA; andrew.s.herbert4.ctr@mail.mil (A.S.H.); samantha.e.zak.ctr@mail.mil (S.E.Z.); jennifer.m.brannan.ctr@mail.mil (J.M.B.); russell.r.bakken.civ@mail.mil (R.R.B.); 2 Unite de Biotechnologie des Anticorps, Institut de Recherche Biomedicale des Armees (IRBA-CRSSA), La Tronche 38516, France; t.pelat@orange.fr (T.P.); pthullier@yahoo.com (P.T.); 3 Institut für Biochemie, Biotechnologie und Bioinformatik, Technische Universität Braunschweig, Braunschweig 38106, Germany; miethe.sem@icloud.com (S.M.); m.hust@tu-bs.de (M.H.); 4 Sutro Biopharma Inc., South San Francisco, CA 94080, USA; asteiner@sutrobio.com (A.R.S.); gyin@sutrobio.com (G.Y.); thallam@sutrobio.com (T.J.H.); aaron.sato@lakepharma.com (A.K.S.)

**Keywords:** Sudan virus, Ebola, antibody, protection, biodefense, cell-free production, phage display

## Abstract

Sudan virus (SUDV) and Ebola viruses (EBOV) are both members of the *Ebolavirus* genus and have been sources of epidemics and outbreaks for several decades. We present here the generation and characterization of cross-reactive antibodies to both SUDV and EBOV, which were produced in a cell-free system and protective against SUDV in mice. A non-human primate, cynomolgus macaque, was immunized with viral-replicon particles expressing the glycoprotein of SUDV-Boniface (8A). Two separate antibody fragment phage display libraries were constructed after four immunogen injections. Both libraries were screened first against the SUDV and a second library was cross-selected against EBOV-Kikwit. Sequencing of 288 selected clones from the two distinct libraries identified 58 clones with distinct V_H_ and V_L_ sequences. Many of these clones were cross-reactive to EBOV and SUDV and able to neutralize SUDV. Three of these recombinant antibodies (X10B1, X10F3, and X10H2) were produced in the scFv-Fc format utilizing a cell-free production system. Mice that were challenged with SUDV-Boniface receiving 100µg of the X10B1/X10H2 scFv-Fc combination 6 and 48-h post-exposure demonstrated partial protection individually and complete protection as a combination. The data herein suggests these antibodies may be promising candidates for further therapeutic development.

## 1. Introduction

Sudan virus (SUDV) along with the other four members of the *Ebolavirus* genus, with *Marburgvirus* and *Cuevavirus,* constitutes the family *Filoviridae* of the order Mononegavirales. SUDV causes severe and highly lethal viral hemorrhagic fevers (VHF) in both non-human primates (NHP) and humans [1]. This class of viruses have the capacity to elicit devastating impact on global health, as was made evident by Ebola virus (EBOV) in the 2014–2016 West Africa outbreak. As with EBOV, the primary transmission of SUDV is through contact with infected bodily fluids from infected humans or animals. SUDV was first identified in an outbreak in South Sudan in 1976 and continues to cause sporadic outbreaks throughout equatorial Africa [2]. All filoviruses are non-segmented, single-stranded negative sense RNA viruses that contain seven or more structural proteins [3]. The transmembrane glycoprotein (GP) is expressed on the viral surface and is the primary facilitating protein of entry into the host cells [4]. The location and abundance of this protein on the virion surface makes it an attractive candidate for the development of protective antibodies.

No therapeutic or vaccine options are currently approved for SUDV, however, several efforts are being pursued for EBOV medical counter measures which include not only monoclonal antibody cocktails [5,6,7,8], but small molecule therapeutics, post-exposure vaccines, and host response interventions [9]. Specific to SUDV, several antibodies have been reported which provide protection against SUDV in rodent models. The first and most effective of these, 16F6, produced by murine hybridoma technology, binds to the GP1 subunit of SUDV GP and has shown complete protection in rodent models [10]. FVM04, a macaque derived monoclonal is able to provide complete protection against EBOV and partial protection to SUDV in a rodent infection model [7]. The ability to identify broadly neutralizing antibodies (bNAbs) across *Ebolavirus* genus has recently been identified from a human survivor [11]. Vaccine candidates have shown varying degrees of success in animal models (reviewed in [12,13,14]). The shared component of all these vaccine candidates was the concept of developing an immune response against GP, which would hopefully lead to the generation of protective antibodies and cellular responses. A combination of approaches utilizing a vaccine program as well as the utilization of immunotherapy and small molecule therapy may be required to respond to all elements present during an outbreak.

We have recently presented the development of macaque derived antibodies to Marburg virus (MARV) utilizing a similar method [15]. In this study, we report the generation, isolation and characterization of high-affinity single chain variable fragments (scFvs) targeting SUDV GP which are able to neutralize and protect individually, and provide combinatorial protection as a two antibody cocktail. Utilizing a cell-free expression methodology, we demonstrate a potential accelerated approach for the production of potential antibody and/or antibody fragments for functional assessment and characterization.

## 2. Materials and Methods

### 2.1. Macaque Immunization

Virus replicon particles (VRPs) on a Venezuelan equine encephalitis virus platform were first developed by Pushko et al. [16]. Filovirus-specific VRPs expressing SUDV GP have been previously shown protection in rodents and NHPs [17]. VRPs expressing SUDV GP were injected intramuscularly (i.m.) into a cynomolgus macaque (*Macaca fascicularis*). The first injection consisted of SUDV GP expressing VRP at a concentration of 1.0 × 10^9^ VRP/mL. Two additional injections were completed at 30 day intervals followed by a final booster (fourth) injection 104 days after the third injection, all at 1.0 × 10^9^ VRP/mL. 

The macaque immunizations were approved by the Institut de Recherche Biomédicale des Armées Ethics committee (Comité d’éthique de l’Institut de Recherche Biomédicale du Service de Santé des Armées, permission date (27,May, 2011)) under authorization no. 2008/03.0 and were performed in accordance with all relevant French laws and ethical guidelines, including, in particular (1) “partie règlementaire du livre II du code rural (Titre I, chapitre IV, section 5, sous-section 3: expérimentation sur l’animal)”; (2) “décret 87-848 du 19-10/1987 relatif aux expériences pratiquées sur les animaux vertébrés modifié par le décret 2001/464 du 29/05/2001”; (3) “arrêté du 29 octobre 1990 relatif aux conditions de l’expérimentation animale pour le Ministère de la Défense”; and (4) “instruction 844/DEF/DCSSA/AST/VET du 9 avril 1991 relative aux conditions de réalisation de l’expérimentation animale”. Animal care procedures complied with the regulations detailed under the Animal Welfare Act and in the *Guide for the Care and Use of Laboratory Animals*. Animals were kept at a constant temperature (22 °C ± 2 °C) and relative humidity (50%), with 12 h of artificial light per day. Animals were anesthetized before the collection of blood or bone marrow by an intramuscular injection of 10 mg/kg ketamine (Imalgene^®^, Merial, Lyon, France). If the animal technicians suspected that the animal was in pain, on the basis of their observations of animal behavior, analgesics were subsequently administered, through a single intramuscular injection of 5 mg/kg flunixine (Finadyne^®^, Schering Plough, Herouville Saint Clair, France) in the days after interventions.

### 2.2. Construction and Screening of the Anti-SUDV Antibody Gene Library

RNA from lymphocytes of the macaque bone marrow was prepared with Tri Reagent (Molecular Research Center Inc., Cincinnati, OH, USA). The isolated RNA was reverse transcribed to cDNA using Superscript II and oligo (dT) (Invitrogen, Carlsbad, CA, USA). Combinations of forward and reverse primers were used to amplify the regions coding for the variable regions VLĸ and V_H_ as previously described [18]. PCR products were pre-cloned in the pGemT vector (Promega, Madison, WI, USA) according to the manufacturer’s instructions, yielding two sub-libraries encoding the heavy chains (Fd fragment) or the κ light chains.

The pGemT cloned PCR products were reamplified using two macaque oligonucleotide primer sets to introduce restriction sites for library cloning as described before [19,20,21]. In brief, the secondary PCRs were carried out for each forward oligonucleotide primers separately to keep the diversity. Each PCR was performed in a volume of 100 µL using 100 ng purified PCR reaction product of the pGemT cloned cDNA, 2.5 U Go Taq polymerase (Promega, Mannheim, Germany), 200 µM dNTPs each, and 200 nM of each oligonucleotide primer for 20 cycles (30 s, 94 °C; 30 s, 57 °C; 30 s, 72 °C), followed by 10 min 72 °C. The PCR products were separated by 1.5% (w/v) agarose gel, cut out and purified using Nucleospin Extract II Kit (Macherey-Nagel, Düren, Germany) according to the manufacturer’s instructions.

The construction of the library was completed in two subsequent steps. First, the PCR products encoding V_L_ were cloned into pHAL35. [22] Second, the VH PCR fragments were cloned. A total of 5 µg pHAL35 and 2 µg V_L_ were digested using 50 U MluI and 50 U NotI (NEB, Frankfurt, Germany) in a 100 µL reaction volume for 2 h at 37 °C. Afterwards, 0.5 U calf intestinal phosphatase (MBI Fermentas, Waltham, MA, USA) was added and incubated for further 30 min. This dephosphorylation step was repeated once. The vector was purified using the Nucleospin Extract II Kit. 270 ng V_L_ were cloned into 1 µg of the dephosporylated pHAL35 using 1 U ligase (Promega, Mannheim, Germany) overnight at 16 °C. The ligation reactions were precipitated with ethanol and sodium acetate and the pellet was washed twice with 70% ethanol. These reactions were electroporated (1.7 kV) in 25 µL XL1-Blue MRF’ (Agilent, Böblingen, Germany). The transformed bacteria were plated onto 2xYT agar plates (Sambrook and Russell, 2001) (25 cm petri dishes) supplemented with 100 µg/mL ampicillin, 20 µg/mL tetracycline, and 100 mM glucose. The colonies were harvested by suspending in 40 mL 2xYT media with a Drigalsky spatula. Plasmids were isolated using the Nucleobond Plasmid Midi Kit (Macherey-Nagel, Düren, Germany) according to the manufacturer’s instructions. Afterwards, 5 µg of each V_L_ chain library as well as 2 µg of the V_H_ fragments were digested using 50 U HindIII (NEB) in a 100 µL reaction volume overnight at 37 °C followed by 50 U SfiI (NEB) for 2.5 h at 50 °C. In total, four transformations were performed and pooled. The harvested bacteria representing the final antibody gene libraries were aliquoted and stored at −80 °C.

### 2.3. Library Packaging

400 mL 2xYT medium supplemented with 100 µg/mL ampicillin and 100 mM glucose were inoculated with the library glycerin stock of the pooled library [23]. The bacteria were grown to O.D.600 = 0.4 − 0.5 at 37 °C and 250 rpm. 25 mL bacteria (~1.25 × 10^10^ bacteria) were infected with 2.5 × 10^11^ hyperphage, incubated at 37 °C for 30 min without shaking, followed by 30 min at 250 rpm [24,25]. The infected cells were harvested by centrifugation for 10 min at 3220× *g* and the pellet was resuspended in 30 mL 2xYT supplemented with 100 µg/mL ampicillin and 50 µg/mL kanamycin, and cultivated over night at 30 °C and 250 rpm. Bacteria cells were pelleted for 10 min at 10,000× *g*. Phage particles in the supernatant were precipitated with one-fifth volume of 20% PEG/2.5 M NaCl solution for 1 h on ice with gentle shaking and pelleted 1 h at 10,000× *g* at 4 °C. The precipitated phage were re-suspended in 10 mL phage dilution buffer (10 mM TrisHCl pH 7.5, 20 mM NaCl, 2 mM EDTA), sterile filtered using a 0.45 µm filter and precipitated again with one-fifth volume of PEG solution for 20 min on ice, and pelleted 30 min at 10,000× *g* at 4 °C. The precipitated phage were re-suspended in 300 µL PBS (phosphate buffered saline) and cell debris was pelleted by additional centrifugation for 5 min at 15,400× *g* at 20 °C. The supernatant containing the scFv phage were stored at 4 °C. The library packaging was analyzed by SDS-PAGE, Western blot and anti-pIII immunostaining as described before [19].

Screening of the library was performed as described elsewhere [15], except that 5, 10, 20, and 40 washes were performed for each successive round of panning. (Supplemental Figure S1) SUDV GP or irradiated whole virus were utilized as the antigens and TBS-Tween 20 0.1% as the washing buffer. The third round of washing from the parental library “D10-RIII”, corresponding to 20 washes was cross-panned to EBOV GP. This library was washed utilizing a single round at 5, 10, or 20 washes in parallel.

### 2.4. Affinity and Cell Based Neutralization 

Affinities were measured by surface plasmon resonance (SPR) utilizing a Biacore-3000 instrument (Biacore, Uppsala, Sweden). The SUDV GP was immobilized at a maximum of 1000 RU on a CM5 chip (Biacore) via amine coupling according to the manufacturer’s instructions. A 30 µL/min flow rate was maintained for the measurement. For each scFv, eight dilutions were prepared in HBS-EP buffer (Biacore) with elution times greater than 1000 s. Following each dilution, the chip was regenerated with 1.5 M glycine buffer (Biacore) run at 10 µL/min for 50 s. For competition Biacore epitope binding, SUDV GP was immobilized at a maximum of 400 RU on a CM5 chip (Biacore) as above. 

Antibody samples, in the scFv format, were titrated in complete MEM supplemented with 10% FBS. Antibody dilutions were added, in decreasing dilutions, to a constant viral titer for 65 PFU per well for a 1 h incubation at 37 °C. Dilutions were plated in triplicate on 6-well plates containing 95–98% confluent Vero E6 cells. After a 1 h incubation at 37 °C, wells were overlaid with 1% agarose in Eagle’s basal medium (EBME) with 10% FBS and 0.1% gentamicin and returned to the incubator for seven days. On day 7, a 1% agarose secondary overlay containing 4% neutral red was added and after one more day at 37 °C, plaques were counted [26].

### 2.5. Cell-Free scFv-Fc Production and Purification

Cell-free expression was carried out in the system previously described [27]. In brief, DNA sequences encoding the four candidate scFv-Fc were synthesized (ATUM; Menlo Park, CA, USA) and cloned into the pYD317 expression vector. Plasmid DNA of these vectors was prepared using Qiagen Maxi Kits per manufacturer’s recommendation and subsequently utilized in cell-free expression. Cell-free expression reactions were performed using the methods previously described by Yin et al. [28] and initial titers assessed by 14C autoradiography. 

Scale-up to 5–10 mg of scFv-Fc for the three lead candidates (X10B1, X10H2, and X10F3) was accomplished by scaling the cell-free reactions to 100 mL. After 16 h of reaction time, the crude cell-free reaction was clarified using centrifugation and the supernatant applied to MabSelect SuRe columns (GE Lifesciences, ‎Chicago, IL, USA) to capture the scFv-Fc. The columns were extensively washed, first with 20 mM sodium phosphate, 150 mM sodium chloride, 0.5% Triton X-100, pH 7.2, and then 20 mM sodium phosphate, pH 7.2. Subsequently, purified scFv-Fc was eluted with 0.1 M sodium citrate pH 3.0 and then dialyzed into storage buffer (DPBS + 5% sucrose).

### 2.6. Murine Protection Studies

Specific pathogen-free six- to eight-week-old male and female INF α/β receptor knockout (IFNAR-/-) mice were utilized (Jackson Laboratory, Bar Harbor, ME, USA) as a model for filovirus infection. Research was conducted under an IACUC approved protocol in compliance with the Animal Welfare Act, PHS Policy, and other Federal statutes and regulations relating to animals and experiments involving animals. The facility where this research was conducted is accredited by the Association for Assessment and Accreditation of Laboratory Animal Care, International and adheres to principles stated in the *Guide for the Care and Use of Laboratory Animals*, National Research Council, 2011. On Day -5 to -2, mice were transferred to a Biosafety Level 4 containment area and challenged by i.p. inoculation utilizing 1000 plaque forming units (PFU) SUDV-Boniface on D0. One-hundred micrograms of total antibody (100 µg for single administration or 50 µg/antibody for the combination groups) was administered intraperitoneally (i.p.) to groups of mice (*n* = 10/gender with *n* = 20/treatment group) as a scFv-Fc fusion, on Days 0 (6 h post) and day 2 (48-h post). Mice were weighed and monitored and once or twice daily upon onset of symptoms for 28 days post infection. Efficacy assessment statistics utilized a Fisher’s exact test. Weight loss was not used as a euthanasia criteria.

Murine challenge studies were conducted under IACUC-approved protocols in compliance with the Animal Welfare Act, PHS Policy, and other applicable federal statutes and regulations relating to animals and experiments involving animals. The facility where these studies was conducted (USAMRIID) is accredited by the Association for Assessment and Accreditation of Laboratory Animal Care, International (AAALAC) and adhere to principles stated in the Guide for the Care and Use of Laboratory Animals, National Research Council, 2011.

## 3. Results

### 3.1. Macaque Immunization and Antibody Generation

A single cynomolgus macaque was intramuscularly (i.m.) immunized with four sequential injections of virus replicon particles (VRP) expressing Sudan GP (Boniface 8A isolate) on the cell surface following viral replication of the complex. The macaque developed increasing anti-GP Ab titers as evaluated by ELISA with a titer of 1:25,000 after the second boost and 1:50,000 following the third. (Supplemental Table S1) Prior to the final injection, a pre-boost bone marrow sample is taken to insure that the RNA response in the bone marrow has been reduced and the final boost will produce a specific signal which identifies strong variable family responses. The final boost was given three months after the third injection and eight days later bone marrow samples were harvested. Bone marrow samples were taken on days 3, 6, 8, 10, 12, 18, and 21. The strongest DNA amplification was observed at the day 10 time point (Supplemental Table S2 and Table S3) before the quantity of the amplified variable gene products decreased. The amplified products of VH1 through VH9 and VL1 through VL7 were combined from day ten collections and cloned into pGemT for the respective construction of κ light chains and Fd sub-libraries.

### 3.2. Library Construction and Isolation of scFvs Specific to SUDV-GP

**Figure 1:**
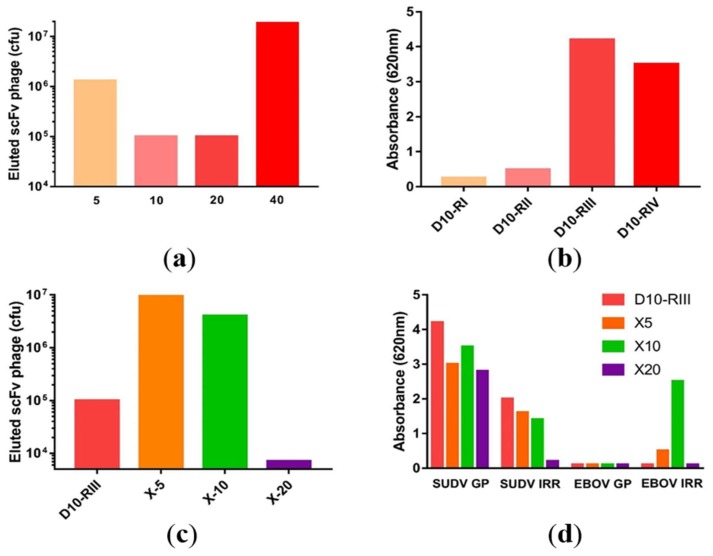
Clones which were reactive to both EBOV and SUDV irradiated virus: (**a**) scFv phage elution titers and (**b**) scFv phage reactivity following consecutive panning rounds for the multi-step library panning. (**c**) Phage elution titers from the third round of the parent library (D10-RIII) with each of the parallel panning rounds to Ebola GP at 5, 10, and 20 washes. (**d**) Reactivity to the whole irradiated antigen (IRR) or glycoprotein (GP) for the parental and cross paining of the SUDV phage against EBOV.

Using the pGemT precloned V-genes, a macaque scFv immune libraries were constructed using pHAL35 [22]. The final library had a size of 1.5 × 10^8^ independent clones. The library underwent a multi-step panning first against SUDV-Boniface GP. Four successive wash rounds at 5, 10, 20, and 40 washes allowed the isolation of 1.5 × 10^8^ phages. Starting from the isolated clones retrieved from the fourth round of panning, 96 clones were randomly hand-picked for sequencing, allowing the isolation of 28 non redundant clones. (Supplemental Figure S1) A parallel panning was also attempted on the whole irradiated virus at a dilution of 1:500 and 1:1000 but these libraries failed after the second round of panning. Given the potential to identify cross reactive antibodies to SUDV and EBOV, the third round of panning of the standard library (RIII) was selected to be crossed panned against EBOV GP based on its phage elution count and high reactivity. The library was panned in parallel to EBOV GP using three stringency protocols of 5, 10, or 20 washes, renamed X5, X10, and X20. The X5 and X10 panning strategies yielded high phage elution counts but only the X10 panning approach resulted in scFv clones which were reactive to both EBOV and SUDV irradiated virus. (Figure 1). Thirty-eight additional individual non-redundant V_H_/V_L_ sequences were isolated from 96 clones that were sequenced specific to the X10 panning strategy. The primary variability of these sequences was mainly located in the V_L_, with limited or no variability in the V_H_.

### 3.3. Antibody Recovery and Characterization

Forty-one non-redundant distinct scFv clones were selected from the two panning strategies; 16 from the standard panning and 25 from the X10 panning. The affinities of each antibody were analyzed for its binding capacity with SUDV GP by surface plasmon resonance (SPR) on Biacore system under standard conditions. The affinities of the anti-SUDV scFv were evaluated and ranged from 300 pM for D10H4 to 81 nM for X10F3 . Of note, several of the X10 antibodies were also reactive to EBOV GP, with improved affinities over SUDV (Table 1). Several clones were unable to produce sufficient quantities of antibody to be further tested and were down-selected.

**Figure 2:**
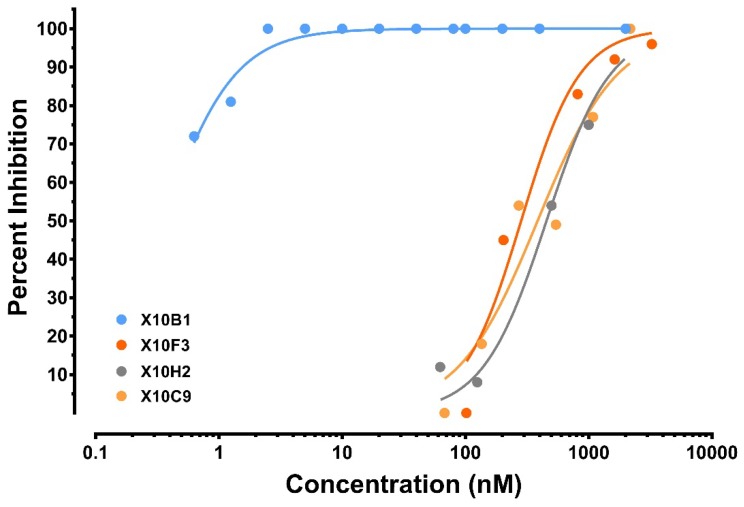
Down-selected antibodies neutralizing activity was evaluated by PRNT. Serially diluted mAbs were mixed in complete MEM supplemented with 10% FBS with SUDV at a constant viral titer of 65 pfu prior to adding in to Vero cells for a 1 h incubation. Wells were overlaid with 1% agarose in Eagle’s basal medium (EBME) with 10% FBS and 0.1% gentamicin and returned to the incubator On day 7, a 1% agarose secondary overlay containing 4% neutral red was added and after one more day at 37 °C, plaques were counted.

In this study, we utilized the plaque reduction and neutralization titer (PRNT) assays to evaluate the neutralization activities of each scFv from the different panning strategies utilizing the SUDV-Boniface. Due to the large number of antibodies, we set a screening cut-off of being able to achieve 80% neutralization (PRNT_80_) at 50 µg/mL scFv against the virus. Nine of the antibody fragments (X10B1, X10B6, X10F3, X10C9, X10G8, X10H2, X20C1, X20D6, and X20H11) achieved PRNT80 at the initial screening concentration and four of them (X10B1, X10F3, X10C9, and X10H2) demonstrated high levels of neutralization (Figure 2).

### 3.4. Cell-Free Production Platform for Rapid scFv-Fc Antibody Production

In an attempt to move as rapidly as possible to a half-life extended format of the four promising scFv candidates and assessment of efficacy in murine models, we elected to convert to an scFv-Fc scaffold, and forgo the time and effort required to ensure conversion to full length mAbs. Also in the interest of rapid production, cell-free expression was chosen as the expression system, as it allows for direct expression of pDNA templates without the need for cell-line development. The four candidate scFvs (X10B1, X10F3, X10C9, and X10H2) were thus converted to scFv-Fcs, gene synthesized, and expressed in an *E. coli* derived cell-free system. 

**Figure 3:**
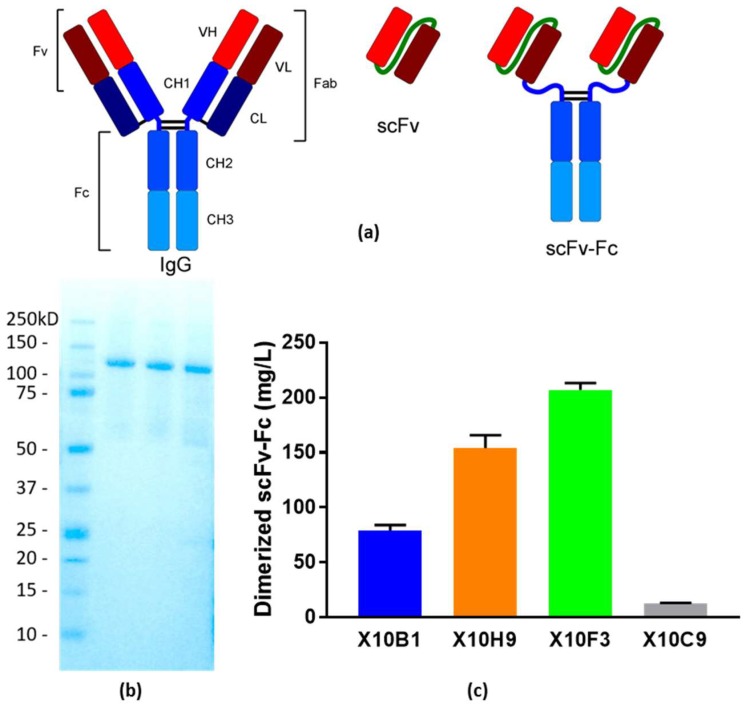
Cell-free expression of candidate scFv-Fc antibody fragments. (**a**) Representation of the structure of the three antibody fragments utilized in this study. The typical IgG format is shown on the left, the scFv format utilized for binding and neutralization in the center, and the scFv-Fc format utilized in neutralization and protection studies on the right. (**b**) Coomassie stained SDS-PAGE Gel of purified scFv-Fcs. Precision Plus Protein™ Standards (BioRad, Hercules, CA, USA); Lane 2: X10F3; Lane 3: X10H2; Lane 4: X10B1. (**c**) Titers from the cell-free expression of the candidate scFv-Fcs.

A range of expression titers across the four candidate scFv-Fcs was observed, with three of them yielding at or above 75 mg/L, while expression of X10C9 was problematic. (Figure 3) Hence, only candidates X10B1, X10H2, and X10F3 were scaled and further assessed for their binding and neutralization. The reactivity of these antibodies to bind to SUDV Boniface GP utilizing a qualitative ELISA were 1:6400 for X10B1; >1:200,000 for X10H2; and 1:100,000 for X10F3. The PRNT_80_ neutralization titers for each of the antibodies were 109 ng/mL for X10B1 and 5 µg/mL for both X10H2 and X10F3. 

### 3.5. In Vivo Murine Protection 

**Figure 4:**
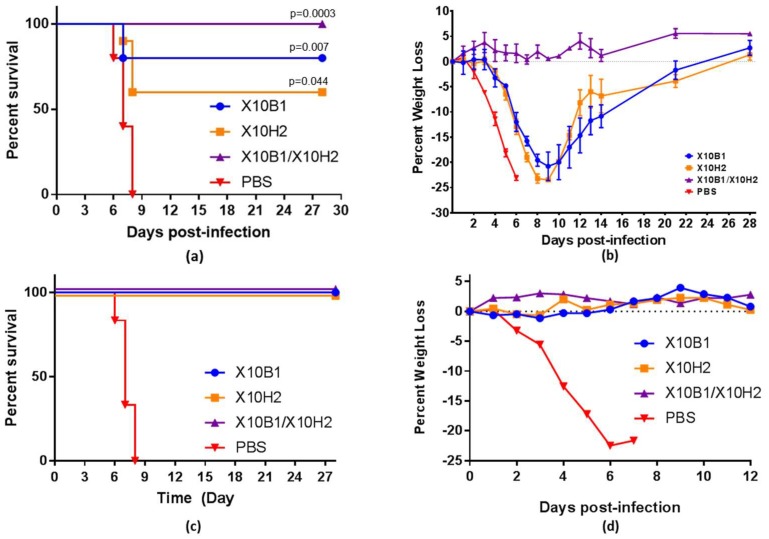
(**a**) Percent survival following the challenge of IFNAR-/- mice administered SUDV GP specific antibodies in the scFv-Fc format. Each mouse (*n* = 10 per antibody group or *n* = 5 for the PBS control) was challenged with 1000 pfu SUDV Boniface on D0; (**b**) weight loss of IFNAR-/- mice administered SUDV GP specific antibodies. Each mouse (*n* = 10 per antibody group) was administered 100 μg of total antibody (100 µg for single administration or 50 µg/antibody for the combination groups) the indicated treatment or PBS (*n* = 5) on days 0 (1 h post) and 2. Mice were challenged with 1000 pfu SUDV Boniface on D0; (**c**) percent survival; and (**d**) weight loss following the re-challenge of IFNAR-/- mice administered SUDV GP specific antibodies. Each mouse (*n* remaining from challenge study group or *n* = 5 for the PBS control) was rechallenged with 1000 pfu SUDV Boniface on D35 of the original study, indicated as D0 above. All *p*-values are assessed against irrelevant controls.

To investigate the in vivo protection, the cell-free production system was scaled to provide sufficient antibody for single and multidose treatments. Two of the selected candidates (X10B1 and X10H2) were produced as scFv-Fc format in sufficient quantities to be tested in the IFN α/β receptor knockout mouse (IFNAR-/-) model challenged with wild-type Sudan Boniface. Standard mouse models, using C57BL/6 or BALB/c, could not be utilized as a mouse adapted as the variant SUDV is not available and the wild-type virus does not cause morbidity or mortality in these strains. However, Brannan et al. identified that IFNAR-/- murine model was susceptible to illness with wild-type SUDV [13]. Mice were challenged with 1000 plaque forming units (pfu) SUDV-Boniface on D0. Treatment groups (*n* = 10) were intraperitoneally (i.p.) administered 100 µg of antibody or PBS (*n* = 5) on day 0 (6 h post) and day 2. X10B1 and X10H2 were able to provide 80% (*p* = 0.007) and 60% (*p* = 0.044) respective protection when administered individually but the weight loss was not statistically significant above controls. (Figure 4a) However, when given as a combination, X10B1 and X10H2 demonstrated a combinatorial response, providing complete protection (*p* = 0.0003) and resulting in no weight loss of the animals. (Figure 4a,b) Thirty-five days after the initial challenge, surviving mice were re-infected with a second injection of 1000 pfu by i.p. and no antibody or PBS treatment. All mice survived (*p* = 0.0003) after the second challenge with no loss in weight, demonstrating that these mice were able to develop their own protective memory immune response (Figure 4c,d).

## 4. Discussion

Previous studies have demonstrated that post-exposure polyclonal antibodies as well as recombinant monoclonal antibodies provide protection against filoviruses in NHP models. Although there is no clear path for the down selection of antibodies against these emerging diseases, we chose an approach which identified high binding affinity to the antigen, neutralization and production capacity in a cell free system, before assessing prototype molecules protective efficacy. In this study, we present the two monoclonal antibodies, developed from NHP immune libraries, and produced in a cell free production system, which are able to elicit combinatorial protective efficacy in murine models. As the 2014–2016 Ebola virus outbreak in western Africa demonstrated, the emergence of a virus could present itself in a population unvaccinated or prepared for such an insult of disease. Given the half-life of current human antibodies and the advancement of Fc-mediated extension motifs, antibody based therapeutics could be utilized not only as therapeutic molecules, but also as a pre-treatment or pre-exposure prophylaxis (PrEP).

In this study, we have demonstrated the ability to select, screen, and produce primate antibodies in a sequence of technologies that could provide a rapid response to viral threats. Utilization of cell-free expression greatly accelerates the production of many potential antibody/antibody fragments for functional assessment and characterization. This work further demonstrates that the down-selection of antibody candidates to infectious diseases should be carefully assessed through in vitro and in vivo assessments. The initial analysis utilizing only the neutralization may not have led to the assessment of X10B1 for in vivo challenge. However, in addition to neutralization, candidates were selected for production in the scFv-Fc format based on the phylogenetic differentiation, neutralization, and the potential for future assessment as cross-protective based on the results demonstrated in Table 1. Fundamentally, the use of a cellular extract for cell-free protein production enables the processes of biomass production and antibody production to be separated. The ribosome-rich extract contains all the necessary components for energy generation, transcription, translation, and antibody assembly, and is agnostic to what protein it is used to produce and can be stockpiled. In this way, moving forward from production of milligram quantities of antibody from DNA within days, as demonstrated here, to rapid cell-free cGMP manufacturing of many kilograms in a few weeks will be feasible [29] and could be particularly impactful in an epidemic emergency response setting. This compares favorably to more traditional cell-based production systems, which would require the creation of either stable cell pools or stable cell lines. Such activities would require roughly two to eight weeks before large scale processes could be developed with newly created stable cells. Conversely, large scale production could be immediately accessible through the cell-free platform process using stockpiled cellular extract as the means for protein production.

These studies identify new therapeutic options against Sudan virus and highlight one possibility for rapid development of antibody based medical countermeasures.

## 5. Patents

An invention disclosure for these antibodies has been filed by USAMRIID through the Department of the Army with a title of “Cross Reactive Antibodies to Sudan and Ebola Virus”. (RIID 18-16)

## Figures and Tables

**Table 1:** Affinity and neutralization screening. Affinities were measured by surface plasmon resonance (SPR) utilizing Biacore utilizing the glycoprotein for each virus coated to the chip. Neutralization screenings were set at 50 ug/mL for each antibody to either SUDV Boniface or EBOV Kikwit and assessed by plaque reduction and neutralization titer (PRNT) assay.

	Affinity (nM)		Neutralization Screen ^1^	
Antibody	SUDV	EBOV	SUDV	EBOV
X10B1	17.3	13.3	+++	+
X10B6	8.3	5.9	+++	-
X10H2	7.0	ND ^2^	+++	+
X10F3	61.0	ND ^2^	+++	+
X10C9	12.0	ND ^2^	+++	-
X10H4	8.5	6.3	+++	-
X10G8	42.0	ND ^2^	+++	ND ^2^
X10H11	20.0	26.1	+++	+
X10H12	8.9	13.1	+	-
X20C3	14.0	8.5	-	-
X20D6	18.7	32.0	+++	ND ^2^
X20F12	14.0	12.4	-	-
X20A4	9.0	5.9	-	ND ^2^
X20A9	29.0	9.0	-	ND ^2^

^1^ +++ Greater than 80%, ++ 50-80%, + 20-50%, - 0-20%; ^2^ ND – Not Determined/Not Tested.

## Supplementary Materials

The following are available online at http://www.mdpi.com/1999-4915/10/6/286/s1. Table S1: RNA Extraction quantities following successive bone marrow sampling. Table S2: Heavy and light chain amplification by RT-PCR. Figure S1: PCR Amplification of VH and VL chains. Figure S2: Schematic demonstrating the phage panning strategy.
