# Supplementary material for: Post-Exposure Protection in Mice against Sudan Virus by a Two Antibody Cocktail
Source: Viruses. 2018 May 26;10(6):286. doi: 10.3390/v10060286 (PMC6024315; doi:10.3390/v10060286)
Supplement: Supplementary file 1 [file viruses-10-00286-s001.docx]

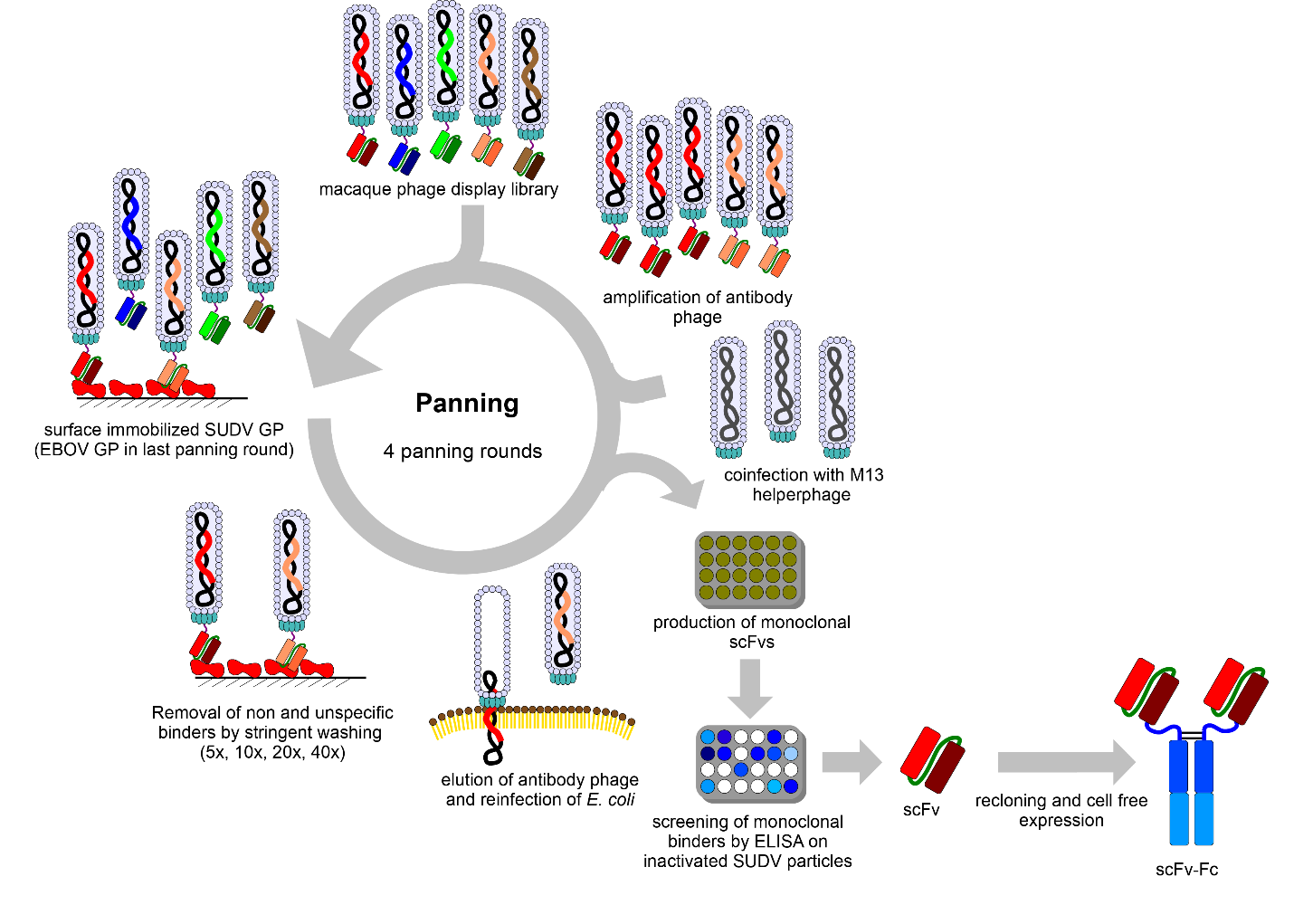


Supplemental Figure 1: Schematic Representation of the panning strategy and subsequent selection and screening on candidate sequences.

Supplemental Table 1: ELISA serum titer response following each successive dose of VRP

Supplemental Table 2: RNA Extraction quantities following successive bone marrow sampling. RNA samples are reflected as solid recovery. Generally, each sample is in 200 µL of ddH2O

Supplemental Table 3: Heavy and light chain amplification by RT-PCR.
